# Supplementary material for: FABP3-mediated membrane lipid saturation alters fluidity and induces ER stress in skeletal muscle with aging
Source: Nat Commun. 2020 Nov 9;11:5661. doi: 10.1038/s41467-020-19501-6 (PMC7653047; doi:10.1038/s41467-020-19501-6)
Supplement: Supplementary file 1 — Supplementary Information [file 41467_2020_19501_MOESM1_ESM.docx]

Supplementary Information for

**FABP3-mediated membrane lipid saturation alters fluidity and induces ER stress in skeletal muscle with aging**

Lee et al.

**Supplementary Fig. 1. Lipid characterization of young and aged TA muscles. a** Pie charts showing the proportion of lipid classes in young and aged muscles (*n*=4 mice per group). **b-g** Major membrane phospholipid species were classified as PC (**b**), PE (**d**), SM (**f**), and LPC (**g**). The proportion of each lipid species in young and aged muscles was normalized to that of total identified lipids. The proportion of PC (**c**) and PE (**e**) species containing the same number of double bonds. Data are presented as means ± S.E.M. Two-tailed unpaired Student’s *t*-test was used. Source data are provided as a Source Data file.

**Supplementary Fig. 2. Lipid characterization of FABP3-overexpressing young TA muscle. a** HA-cherry-FABP3 or HA-cherry was transfected into young TA muscle (*n*=3 mice per group). At 5 days after transfection, FABP3 expression was confirmed by immunoblot analysis using anti-FABP3 or HA antibodies. GAPDH was used as a loading control. **b** Pie charts showing lipid class proportions in FABP3-overexpressing young and control muscles (*n*=4 mice per group). **c-h** Major membrane phospholipid species were classified as PC (**c**), PE (**e**), SM (**g**), and LPC (**h**). The proportion of each lipid species in FABP3-overexpressing young and control muscles was normalized to that of total identified lipids. Proportion of PC (**d**) and PE (**f**) species containing the same number of double bonds. Data are presented as means ± S.E.M. Two-tailed unpaired Student’s *t*-test was used. Source data are provided as a Source Data file.

**Supplementary Fig. 3. FABP3 displays a unique ER stress pattern primarily via PERK-eIFα-dependent protein synthesis.** FABP3 expression was induced in fully differentiated C2C12 mnyotubes harboring Cre-inducible FABP3 constructs by Cre recombinase-carrying adenovirus (Ad-Cre). **a** Immunoblot analysis of FABP3 expression, PERK and eIF2α phosphorylation and puromycin incorporation at 12, 24, 36, 48 and 72 h post-infection with Ad-Cre. **b-i** C2C12 myotubes were infected with Ad-Cre for 72 h. Palmitate was added for 12 h as an ER stress inducer positive control. **b** Transcript levels of *ATF4* and *Chop*, target genes of eIF2α, were measured using qRT-PCR. The results were normalized to *36B4* mRNA levels. **c** The effect of PERK inhibitor (GSK2606414) on FABP3-induced ER stress. FABP3-overexpressing myotubes were treated with 1 μM GSK2606414 or vehicle for 12h. Immunoblot analysis of PERK and eIF2α phosphorylation and puromycin incorporation. **d** Immunoblot analysis of unfolded protein response indicators such as IRE1α phosphorylation and ATF6 cleavage in FABP3-overexpressing or palmitate-treated myotube. **e** Transcript levels of genes involved unfolding protein response such as *XBP1-s, Gr78/Bip, Erdj4*, and *Edem*. The results were normalized to *36B4* mRNA levels. **f, g, i** Immunoblot analysis of IRE-1α downstream effectors such as JNK, p65, and SEK (**f**) as well as AKT-GSK-3β (**g**) and mTOR signaling (**g**), and autophagy markers (**i**) in FABP3-overexpressing or palmitate-treated myotubes. **h** Immunoblot analysis of mTOR signaling in FABP3-overexporessing and control TA muscles (*n*=3 mice per group). **j** Representative images (left) of FABP3-overexpressing myotubes. Green, MyHC; blue, DAPI. Scale bar, 100 μm. Quantification (right) of myotube diameter. **k** Transcript levels of *Atrogin-1* and *MuRF1.* The results were normalized to *36B4* mRNA levels. Data are presented as means ± S.E.M. *n*=3 independent experiments. Two-tailed unpaired Student’s *t*-test was used. Source data are provided as a Source Data file.

**Supplementary Fig. 4. Transient FABP3 overexpression in TA muscles does not affect muscle mass.** HA-cherry-FABP3 or HA-cherry was transfected into young TA muscle. At 5 days after transfection, TA muscle mass was measured and normalized to body weight (*n*=10). Data are presented as means ± S.E.M. Two-tailed unpaired Student’s *t*-test was used. Source data are provided as a Source Data file.

**Supplementary Fig. 5. Lipid characterization of FABP3 knockdown in aged TA muscle. a** TA muscles from aged mice were infected with Ad-shFABP3 virus or Ad-shControl (*n*=3 mice per group). *FABP3* transcript levels were measured and normalized to *36B4*. **b** Pie charts showing lipid class proportions in FABP3 knockdown aged and control muscles. **c-h** Major membrane phospholipid species were classified as PC (**c**), PE (**e**), SM (**g**), and LPC (**h**). The proportion of each lipid species in FABP3-knockdown aged and control muscles was normalized to that of total identified lipid. The proportion of PC (**d**) and PE (**f**) species containing the same number of double bonds. **i** Principal component plot of the lipidomic profiles for the indicated muscle samples. Data are presented as means ± S.E.M. Two-tailed unpaired Student’s *t*-test was used. Source data are provided as a Source Data file.

**Supplementary Fig. 6. FABP3 does not regulate gene expression of fatty acid desaturases and elongases.** Transcript levels of fatty acid desaturases (**a**) and elongases (**b**) in young versus aged muscles, and FABP3-overexpressing or knockdown muscles were measured using qRT-PCR. The results were normalized to *36B4* mRNA levels (*n* = 6). Data are presented as means ± S.E.M. Two-tailed unpaired Student’s *t*-test was used. Source data are provided as a Source Data file.

**Supplementary Table. Percentages of lipid classes in young versus aged muscles and in FABP3-overexpressing or knockdown muscles.**

| Lipid class | Young (%) | FABP3-overexpressing young muscle (%) | Aged (%) | FABP3-knockdown aged muscle (%) |
| --- | --- | --- | --- | --- |
| Acylcarnitine | 0.21 ± 0.07 | 0.25 ± 0.07 | 0.48 ± 0.22 | 0.42 ± 0.24 |
| Ceramide | 0.16 ± 0.02 | 0.19 ± 0.00 | 0.29 ± 0.03 | 0.25 ± 0.02 |
| Diacylglycerol | 1.19 ± 0.12 | 1.27 ± 0.17 | 0.71 ± 0.08 | 0.69 ± 0.03 |
| Glucosylceramide | 0.18 ± 0.02 | 0.24 ± 0.01 | 0.65 ± 0.14 | 0.31 ± 0.04 |
| Lysophosphatidylcholine | 1.13 ± 0.10 | 1.61 ± 0.21 | 3.15 ± 0.25 | 1.70 ± 0.15 |
| Lysophosphatidylethanolamine | 0.01 ± 0.00 | 0.04 ± 0.01 | 0.08 ± 0.01 | 0.04 ± 0.00 |
| Phosphatidylcholine | 75.58 ± 0.52 | 74.86 ± 0.27 | 72.21 ± 1.01 | 77.07 ± 0.32 |
| Phosphatidylethanolamine | 11.65 ± 0.43 | 11.12 ± 0.33 | 9.41 ± 0.61 | 10.60 ± 0.57 |
| Phosphatidylserine | 0.37 ± 0.02 | 0.41 ± 0.03 | 0.60 ± 0.10 | 0.42 ± 0.02 |
| Sphingomyelin | 2.91 ± 0.13 | 3.77 ± 0.24 | 6.91 ± 0.58 | 3.76 ± 0.23 |
| Triacylglycerol | 5.60 ± 0.42 | 5.08 ± 0.35 | 4.16 ± 0.58 | 3.72 ± 0.63 |
| Free fatty acid | 0.20 ± 0.03 | 0.30 ± 0.04 | 0.31 ± 0.09 | 0.14 ± 0.05 |
| Phosphatidylinositol | 0.77 ± 0.01 | 0.83 ± 0.03 | 0.87 ± 0.09 | 1.02 ± 0.07 |

Data are means ± S.D. (*n*=4 mice per group).
