# Supplementary material for: FABP3-mediated membrane lipid saturation alters fluidity and induces ER stress in skeletal muscle with aging
Source: Nat Commun. 2020 Nov 9;11:5661. doi: 10.1038/s41467-020-19501-6 (PMC7653047; doi:10.1038/s41467-020-19501-6)
Supplement: Supplementary file 3 — Description of Additional Supplementary File [file 41467_2020_19501_MOESM3_ESM.docx]

**Description of Additional Supplementary Files**

File Name: Supplementary Data

Description: Lipidomic data for young versus aged, and FABP3-overexpressing or knockdown muscles.
