# Supplementary material for: FABP3-mediated membrane lipid saturation alters fluidity and induces ER stress in skeletal muscle with aging
Source: Nat Commun. 2020 Nov 9;11:5661. doi: 10.1038/s41467-020-19501-6 (PMC7653047; doi:10.1038/s41467-020-19501-6)
Supplement: Supplementary file 5 — Reporting Summary [file 41467_2020_19501_MOESM5_ESM.pdf]

nature research

Corresponding author(s): Ki-Sun Kwon and Kwang-Pyo Lee

Last updated by author(s): Aug 29, 2020

Reporting Summary

Nature Research wishes to improve the reproducibility of the work that we publish. This form provides structure for consistency and transparency in reporting. For further information on Nature Research policies, see our Editorial Policies and the Editorial Policy Checklist.

Statistics

For all statistical analyses, confirm that the following items are present in the figure legend, table legend, main text, or Methods section.

n/a	Confirmed	
☐	☒	The exact sample size (*n*) for each experimental group/condition, given as a discrete number and unit of measurement
☐	☒	A statement on whether measurements were taken from distinct samples or whether the same sample was measured repeatedly
☐	☒	The statistical test(s) used AND whether they are one- or two-sided<br>*Only common tests should be described solely by name; describe more complex techniques in the Methods section.*
☒	☐	A description of all covariates tested
☐	☒	A description of any assumptions or corrections, such as tests of normality and adjustment for multiple comparisons
☐	☒	A full description of the statistical parameters including central tendency (e.g. means) or other basic estimates (e.g. regression coefficient) AND variation (e.g. standard deviation) or associated estimates of uncertainty (e.g. confidence intervals)
☐	☒	For null hypothesis testing, the test statistic (e.g. *F*, *t*, *r*) with confidence intervals, effect sizes, degrees of freedom and *P* value noted<br>*Give P values as exact values whenever suitable.*
☒	☐	For Bayesian analysis, information on the choice of priors and Markov chain Monte Carlo settings
☒	☐	For hierarchical and complex designs, identification of the appropriate level for tests and full reporting of outcomes
☐	☒	Estimates of effect sizes (e.g. Cohen's *d*, Pearson's *r*), indicating how they were calculated

Our web collection on statistics for biologists contains articles on many of the points above.

Software and code

Policy information about availability of computer code

Data collection	NIS elements 4.30, Zen 2.6 software (Zeiss), Markerview 1.3 (SCIEX), StepOne software v2.3 (Applied Biosystems), LabChart Pro v8, ibright 1500 (filmware 1.2.5)
Data analysis	ImageJ 1.52a, GraphPad Prism 8.4.2, Excel 2016, Cluster 3.0, Rstudio 3.5.2, LabChart Pro v8

For manuscripts utilizing custom algorithms or software that are central to the research but not yet described in published literature, software must be made available to editors and reviewers. We strongly encourage code deposition in a community repository (e.g. GitHub). See the Nature Research guidelines for submitting code & software for further information.

Data

Policy information about availability of data

All manuscripts must include a data availability statement. This statement should provide the following information, where applicable:

- Accession codes, unique identifiers, or web links for publicly available datasets
- A list of figures that have associated raw data
- A description of any restrictions on data availability

All data supporting the findings of this study are available within the paper and its supplementary information files. Source data are provided with this paper.

Field-specific reporting

Please select the one below that is the best fit for your research. If you are not sure, read the appropriate sections before making your selection.

☒ Life sciences ☐ Behavioural & social sciences ☐ Ecological, evolutionary & environmental sciences

For a reference copy of the document with all sections, see nature.com/documents/nr-reporting-summary-flat.pdf

Life sciences study design

All studies must disclose on these points even when the disclosure is negative.

Sample size	No statistical method was used to pre-determine the sample size, but the sample sizes were chosen based on previous publications, which were sufficient for statistical analysis.
Data exclusions	None
Replication	The experimental results were reproduced, and the replication numbers are described in the corresponding figure legends.
Randomization	Animals were randomly allocated for each group.<br>Six different fields were randomly selected for measurement of the cross-sectional area and the myotube diameter using microscope imaging software (NIS-Elements, Nilon).<br>At least 5 or more myotubes were randomly selected for FRAP analysis using confocal microscope equipped with a live cell chamber and Zen software (Zeiss)
Blinding	Lipidomic analyses were performed by an outside lab blinded to the identity of the samples.<br>Most mouse data including muscle weight, force measurement, and microscopy were performed blindly and analysis was done in a non-blind fashion.<br>Other cellular work were not performed in a blinding manner.

Reporting for specific materials, systems and methods

We require information from authors about some types of materials, experimental systems and methods used in many studies. Here, indicate whether each material, system or method listed is relevant to your study. If you are not sure if a list item applies to your research, read the appropriate section before selecting a response.

Materials & experimental systems

n/a	Involved in the study	
☐	☒	Antibodies
☐	☒	Eukaryotic cell lines
☒	☐	Palaeontology and archaeology
☐	☒	Animals and other organisms
☒	☐	Human research participants
☒	☐	Clinical data
☒	☐	Dual use research of concern

Methods

n/a	Involved in the study	
☒	☐	ChIP-seq
☒	☐	Flow cytometry
☒	☐	MRI-based neuroimaging

Antibodies

Antibodies used	primary antibodies:<br>4EBP1 (# 9165), ATG5 (#12994), ATG7 (#8558), ATG12 (#4180), ATG16L (#8089), Beclin1 (#3495), eIF2 alpha (#9722), IRE1 alpha (#3294), mTOR (#2983), p65 (#6956), PERK (#3192), S6K (#9202), SEK (#9152), SQSTM1 (#8025), phospho-4EBP1 (#9459), phospho-AKT S473 (#9271), phospho-GSK-3 beta (#9327), phospho-mTOR (#5536), phospho-eIF2 alpha (#9721), phospho-PERK (#3179), phospho-JNK (#9251), phospho-SEK (#9151), phospho-S6K (#9206) from Cell Signaling<br>phospho-IRE1 alpha (ab48187) from Abcam<br>HA (sc-805), AKT (sc-1618), ATF6 (sc-222799), GSK-3 beta (sc-7291), FABP3 (sc-58274), and MYH (B-5,sc-376157) from Santa Cruz Biotechnology<br>JNK (51-1570) from BD bioscience<br>anti-puromycin (PMY-2A4) from DSHB<br>anti-laminin (L9393) from Sigma-Aldrich<br>GAPDH was developed in our laboratory<br>Secondary antibodies:<br>Goat Anti-Rabbit IgG cross absorbed, FITC (F2765), Goat Anti-Mouse IgG peroxidase conjugated (31430), Goat Anti-Rabbit IgG peroxidase conjugated (31460), and Goat Anti-Mouse IgG1 cross absorbed, Alexa Fluor 488 (#A-21121) from ThermoFisher
Validation	All primary antibodies, except GAPDH, used in this study were validated by the manufacturer company. Validation data / citation can

Validation

be found on the manufacture website by searching the antibody catalog number provided in materials and methods section of our manuscript.
Anti-GAPDH antibody was raised against the following amino acids sequence:
GKVKVGVNGFGRIGRLVTRAAFNSGKVDIVAINDPFIDLNYMVYMFQYDSTHGKFHGTVKAENGKLVIN
GNPITIFQERDPSKIKWGDAGAEYVVESTGVFTTMEKAGAHLQGGAKRVIISAPSADAPMFVMGVNHEKY
DNSLKIISNASCTTNCLAPLAKVIHDNFGIVEGLMTTVHAITATQKTVDGPSGKLWRDGRGALQNIIPAS
TGAAKAVGKVIPELNGKLTGMAFRVPTANVSVVDLTCRLEKPAKYDDIKKVVKQASEGPLKGILGYTEHQ
VVSSDFNSDTHSSTFDAGAGIALNDHFVKLISWYDNEFGYSNRVVDLMAHMASKE.
We have compared specificity of this antibody to previously validated antibody to validate.
GAPDH antibody has been used in many previously publication (Sul et al., Human Molecular Genetics, 2013, Choi et al., Aging, 2016).

Eukaryotic cell lines

Policy information about cell lines

Cell line source(s)

C2C12 cell lines were obtained from ATCC.

Authentication

None of the lines used were authenticated.

Mycoplasma contamination

All cell lines were confirmed to be negative for mycoplasma contamination.

Commonly misidentified lines
(See ICLAC register)

None

Animals and other organisms

Policy information about studies involving animals; ARRIVE guidelines recommended for reporting animal research

Laboratory animals

Young (3 month-old) and old (22-24 month old) male C57BL/6 mice were purchased from the laboratory Animal Resource Center (KRIBB). The mice were fed standard chow (Teklad F6 Rodent Diet 8664, Harlan Teklad, Indianapolis, IN), and housed under controlled temperature at 22-24 °C and a 12 h light/12 h dark cycle with a humidity between 40-60%.

Wild animals

None

Field-collected samples

None

Ethics oversight

The Animal Care and Use Committee of the Korea Research Institute of Bioscience and Biotechnology (KRIBB)

Note that full information on the approval of the study protocol must also be provided in the manuscript.
